# Structural basis of kinesin-1 autoinhibition and its control of microtubule-based motility

**DOI:** 10.1126/sciadv.aeg1267

**Published:** 2026-07-15

**Authors:** Md Ashaduzzaman, Yuqi Tang, Kyoko Okada, Stephen D. Fried, Richard J. Mckenney, Jawdat Al-Bassam

**Affiliations:** ^1^ Department of Molecular Cellular Biology, University of California, Davis, CA, USA.; ^2^ Department of Chemistry, Johns Hopkins University, Baltimore, MD, USA.; ^3^ T. C. Jenkins Department of Biophysics, Johns Hopkins University, Baltimore, MD, USA.

## Abstract

Kinesin-1 was the first microtubule motor identified, responsible for anterograde transport of diverse cargo in eukaryotic cells. Defects or misregulation of kinesin-1 is linked to multiple neurological disorders, and various pathogens exploit kinesin-1 to transport their cargo. In the absence of cargo, kinesin-1 adopts a compact autoinhibited conformation to enable its spatiotemporal regulation and prevent futile energy consumption. Despite its importance, the structural mechanisms for kinesin-1 autoinhibition and activation remain poorly understood. Here, we report the cryo–electron microscopy structure of the autoinhibited kinesin-1 heterotetramer and validate it using cross-linking mass spectrometry. The structure reveals a 36-nanometer particle in which the kinesin heavy chains (KHCs) adopt a head-to-tail configuration, stabilized by asymmetrically arranged kinesin light chain (KLC) tetratricopeptide repeat (TPR) domains that bind across folded KHC coiled coils and in between the KHC motor domains. This architecture inhibits kinesin motility by constraining the dimeric motor domains in a configuration that is incompatible with processive motility. In addition, the structure shows that the KLC carboxyl-terminal helices occlude the TPR cargo-binding interfaces, revealing a second layer of autoinhibition that directly blocks cargo engagement. Functional studies and structural modeling suggest that binding of regulatory factors, such as MAP7D3, competes with intramolecular KHC coiled-coil interactions, resulting in the unfurling of the autoinhibited structure and activating motor motility. These findings provide a molecular framework for understanding kinesin-1 regulation and its implications for intracellular transport.

## Teaser

Cryo-EM reveals the structural basis of kinesin-1 autoinhibition and its activation by MAP7D3.

## INTRODUCTION

Kinesin-1 (hereafter “kinesin”) is the founding member of a superfamily of motor proteins essential for intracellular transport along microtubule (MT) (*1*, *2*). Since its discovery in axonal transport studies, 45 genes encoding kinesin-related proteins have been identified in humans (*2*). As the most ubiquitous MT-based anterograde motor in eukaryotes, kinesin transports a diverse range of cargos, including mRNA, organelles such as mitochondria and lysosomes, and nuclei, toward MT plus-ends (*3*–*6*). While its hand-over-hand processive motility mechanism is well understood from studies of truncated constructs (*7*, *8*), how the full-length motor is precisely regulated remains a central question.

In cells, kinesin exists predominantly as a heterotetramer composed of two kinesin heavy chains (KHCs) from one of three human isoforms (KIF5B, KIF5C, and KIF5A) and two kinesin light chains (KLCs) from one of four human isoforms of KLC1 to KLC4 (*9*). Each KHC is composed of a highly conserved N-terminal motor domain, followed by segments of conserved coiled coils (CC0, CC1^KHC^, CC2^KHC^, CC3, and CC4) and terminating in C-terminal tail domains of distinct lengths (*10*–*12*). Each KLC is composed of N-terminal coiled coils (CC1^KLC^ and CC2^KLC^) connected to cargo-binding tetratricopeptide repeat (TPR) domains via a conserved helical linker region (*13*, *14*). The C termini of KLCs contain variable sequence extensions that are subject to extensive alternative splicing (*15*–*17*). This structural complexity provides multiple points for cellular regulation, from autoinhibition via the KHC tail to cargo-dependent activation through the KLCs (*18*, *19*).

**Fig. 1.**
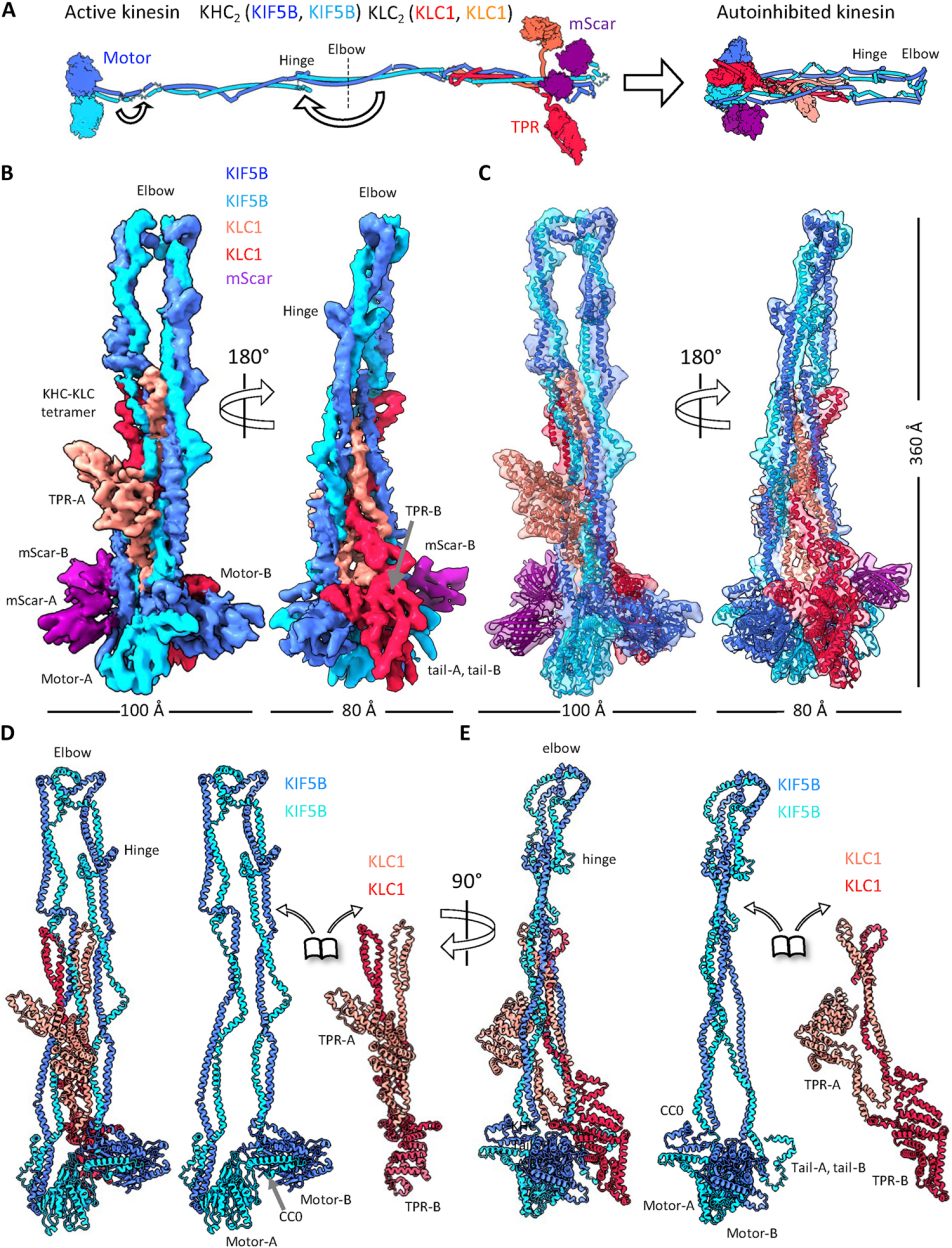
Cryo-EM structure of the autoinhibited kinesin KIF5B-KLC1 reveals the organization of the heterotetramer and the asymmetric subunit interactions in the lambda particle. (**A**) A schematic conceptual illustration showing the linear organization of kinesin heterotetramer with its KHCs (blue and cyan) and KLCs (red and orange). Arrows refer to the conformational folding events leading to the autoinhibited state structure described in this work. (**B**) Two 180° rotated views of a segmented kinesin KIF5B-mScarlet-KLC1 heterotetrametric cryo-EM map. The KHC subunits, KHC-A and KHC-B, are colored blue and cyan, while the KLC subunits, KLC-A and KLC-B, are colored red and salmon. Folded domain densities for the mScarlet (mScar-A and mScar-B) proteins fused on the KHC C termini are colored purple, and KHC motor domains (motor-A and motor-B) and KLC-TPR domains (TPR-A and TPR-B) are also marked. (**C**) Two 90° rotated views of the modeled (shown in ribbon) and segmented transparent kinesin KIF5B-mScarlet KLC1 heterotetramer cryo-EM map (transparent), colored as shown in (A). (**D**) Left: A ribbon model for KIF5B-KLC1 heterotetramer showing the asymmetric KLC-TPR domain interactions with KHC subunits without the mScarlet. Right: A dissociated view showing the isolated KLC (red and orange) and KHC (blue and cyan) dimeric subunits matching the orientation with left full heterotetramer model. (**E**) A 90° rotated view of ribbon models shown in (D).

The kinesin heterotetramer complex intrinsically folds into a compact, autoinhibited conformation (Fig. 1A), a state first inferred from hydrodynamic studies showing that the native particle is roughly half the length of the motile motor (*14*, *20*, *21*). More recently, low-resolution structures from negative-stain electron microscopy (EM) and cross-linking mass spectrometry (XL-MS) visualized this “lambda”-shaped particle (*22*–*24*). Nevertheless, the precise placement of individual KHC and KLC domains within this assembly has remained unclear.

Beyond suppressing motility, the autoinhibited kinesin state must also control cargo engagement. Kinesin binds a vast array of cargoes through two main hubs: The KLC-TPR domains recruit many reported kinesin cargoes using short conserved peptides found within cargo-adapter proteins that bind the concave interior interface of the crescent-shaped TPR domain (*25*, *26*). The KHC C-terminal tail regions bind cargo through adaptors such as c-Jun N-terminal kinase-interacting protein 1 (JIP1), JIP3, tropomyosin and Trafficking of kinesin (TRAK) proteins (*27*–*29*). These adaptors regulate interactions with protein networks including Rab guanosine triphosphatases and kinases that promote signaling-mediated activation of kinesin transport (*5*, *30*). For proper regulation, these cargo-binding sites must be inaccessible in the inhibited state to prevent premature cargo loading and motor activation. However, it has remained unclear how the KLC-TPR and KHC C-terminal domains are configured within the autoinhibited complex and how their functions are regulated.

Decades of research have provided key but sometimes paradoxical clues about the kinesin activation mechanism. The KHC C-terminal tail, containing a conserved isoleucine-alanine-lysine (IAK) motif, is a potent inhibitor of motor activity (*18*, *23*, *31*). However, while tail truncation produces a constitutively active motor, mutation of the IAK motif alone appears insufficient to fully activate processivity in the full-length complex (*22*, *32*). A crystal structure of the kinesin motor domains with an isolated IAK-containing peptide reveals a 1:2 tail-to-motor complex, in which the peptide binds between the dimeric motor domains (*33*). However, studies that mutated the IAK motif in the full-length heterotetramer show that while the motif is required to repress MT binding, its removal is insufficient to strongly activate processive motility (*22*, *32*). This suggests that other factors are required, a role now largely attributed to cargo adapter proteins that bind to the KLCs (*26*, *32*), and MT-associated protein 7 (MAP7) family proteins (*32*, *34*, *35*). MAP7 family proteins have emerged as key regulators of kinesin activity both in vitro and in cells (*32*, *34*–*37*). MAP7 proteins markedly enhance kinesin’s MT association and are required for its activity in cells (*34*, *35*, *38*). This has led to a model of sequential activation, where MT-bound MAP7 first recruits and activates the motor, which, in turn, unmasks the KLCs for cargo binding (*38*). While this model explains many observations, the structural basis for this sequence of events—how the complex physically unfurls to release both motor and cargo inhibition—has remained a mystery.

To help resolve this, we determined a 5.6- to 8.6-Å cryo-EM structure of the autoinhibited human kinesin heterotetramer complex (KIF5B-KLC1). Our structure reveals a notable asymmetry in how KLC1 organizes the complex, in contrast to the symmetrically intramolecularly folded KIF5B. One KLC1 TPR domain binds across the folded KIF5B coiled coils, while the other binds in between the KIF5B tail and motor domains, rendering them incompatible with processive motility. Furthermore, we uncover a previously unknown mechanism of cargo-binding autoinhibition, in which C-terminal KLC helices fold back to sterically block the cargo-binding groove of the TPR domains. Together, these findings provide a comprehensive structural blueprint for how kinesin coregulates its motor and cargo-binding functions, establishing a physical framework for its sequential activation using MAP7D3.

## RESULTS

### Cryo-EM structure of kinesin in the autoinhibited state

We purified recombinant human kinesin heterotetramer KIF5B-mScarlet and KLC1, as previously described (*32*), and optimized mild cross-linking conditions using a modified sucrose density GraFix approach (fig. S1, A and B) (*39*). This process was essential to stabilize the autoinhibited kinesin particles before transfer to conditions compatible with cryo-EM imaging. The fusion of mScarlet onto the KIF5B C terminus via a short linker has no impact on kinesin autoinhibition or activation by MAP7 and cargo (*32*, *38*). Mass photometry of sucrose density gradient-purified fractions enabled the separation of the single heterotetramer (KHC_2_KLC_2_) population from KHC_4_KLC_4_ heterotetramers (fig. S1, C to F). Extensive grid screening and vitrification yielded clean distributions of ~40-nm single closed kinesin particles. A subset of particles adopts partially or fully open, V-shaped conformations ~70 nm in length, consistent with hinge-like opening upon release from autoinhibition. These observations support a key role for cross-linking in stabilizing the autoinhibited state (fig. S2).

Full-size particles were only visible under relatively thick ice conditions, limiting the attainable resolution of our final cryo-EM reconstructions (fig. S2). Initial two-dimensional (2D) classification revealed predominantly closed kinesin particles, with very few particles in the open conformation. 2D classification also revealed flexibility in several regions of the closed 40-nm kinesin particle, particularly in its thinner coiled-coil regions (fig. S2). Iterative 3D classification yielded a curated particle dataset and a 12-Å consensus kinesin map in which the folded coiled coils, KLC-TPR domains, mScarlet fluorescent proteins domains fused onto the KIF5B tails, and KHC motor domains can be confidently identified by shape (fig. S3, top).

To improve map quality and resolution across different regions, we subdivided the kinesin particle into four overlapping regions and performed masked classification and refinement, yielding regional resolutions ranging from 5.6 to 8.6 Å (see Materials and Methods and fig. S3). Nearly 80% of the kinesin particles showed flexibility in a density attributed to one KHC motor domain that interfaces between the KLC-TPR domain and KHC tail domains (fig. S2, bottom, and movie S1). We refined two regions that include each of two KHC motor domains independently while bound to neighboring elements, to improve their local density (fig. S3 and movie S1). The refined subregional maps contained extensively overlapping features and comparable map qualities, allowing the clear alignment of all subregional maps to obtain a consensus map at 5.6- to 8.6-Å resolution (figs. S3 and S4, A to F, and movie S1). The merged map of the full kinesin particle revealed clear α-helical secondary structure elements throughout, allowing model building of the kinesin assembled KHC and KLC coiled coils, and folded domains in their heterotetrametric autoinhibited organization using either experimentally determined structures or AlphaFold3 models (Fig. 1, B and C, and fig. S4).

To document our modeling approach, we present a step-by-step workflow for placing KHC and KLC structural regions into the full kinesin-1 cryo-EM density map (figs. S5 and S6). For each region, we compare the AlphaFold3-predicted domain structures (figs. S5, left, and S6, left) with the final fitted models after manual rebuilding and placement into the corresponding cryo-EM density (figs. S5, right, and S6, right). For the KHC regions (fig. S5), this process illustrates three types of structural modification in coiled coils: relaxation of the superhelical coiled-coil twist (fig. S5B), introduction of two right-angle bends that form a characteristic elbow configuration (fig. S5C), and compression of coiled-coil supertwist (fig. S5D). The dimeric mScarlet domains were unambiguously identified as paired β barrel densities in the cryo-EM map; their modeling provided fixed positional landmarks that enabled confident placement for model building of the flanking dimeric KHC C-terminal tail regions (fig. S5E). For the KLC regions (fig. S6), following placement of the TPR domains, additional helical density was identified and modeled as the C-terminal region of the KLC including the α-helical H1-H2 linker, the SC (self-cargo) region, and the H3 C-terminal extension (fig. S6, B and C). Asymmetry in the linker regions connecting the KLC coiled coils to the TPR domains was also evident and modeled accordingly (fig. S6D). The final experimentally derived model encompasses all KHC and KLC regions, with the exception of the KLC N-terminal ~20 residues, which were not resolved in the density maps.

### Overall organization of autoinhibited kinesin structure

The cryo-EM structure reveals a 36-nm comet-shaped lambda (*23*) particle in which the symmetrical KHC dimer folds back on itself at the “elbow,” creating a turnaround zone for the second half of the KHCs (Fig. 1, B and C, and movie S2). The two KLCs bind the KHC via their N-terminal coiled-coil regions but adopt asymmetrically arranged TPR domains: One TPR (termed TPR-A) stabilizes the folded coiled-coil interface, while the other (termed TPR-B) binds in between the motor domains and tails (Fig. 1, D and E). The KHC coiled coil–containing regions fold back at the elbow junction to form the lambda particle (*23*), bringing the N-terminal KHC motor domains into proximity with the C-terminal KHC tails (Fig. 1, D and E). Both KHC tails are ordered, while fused via short linker sequences, to two ordered mScarlet fluorescent β barrel fold domains (termed mScar-A and mScar-B) (Fig. 1, B and C).

**Fig. 2.**
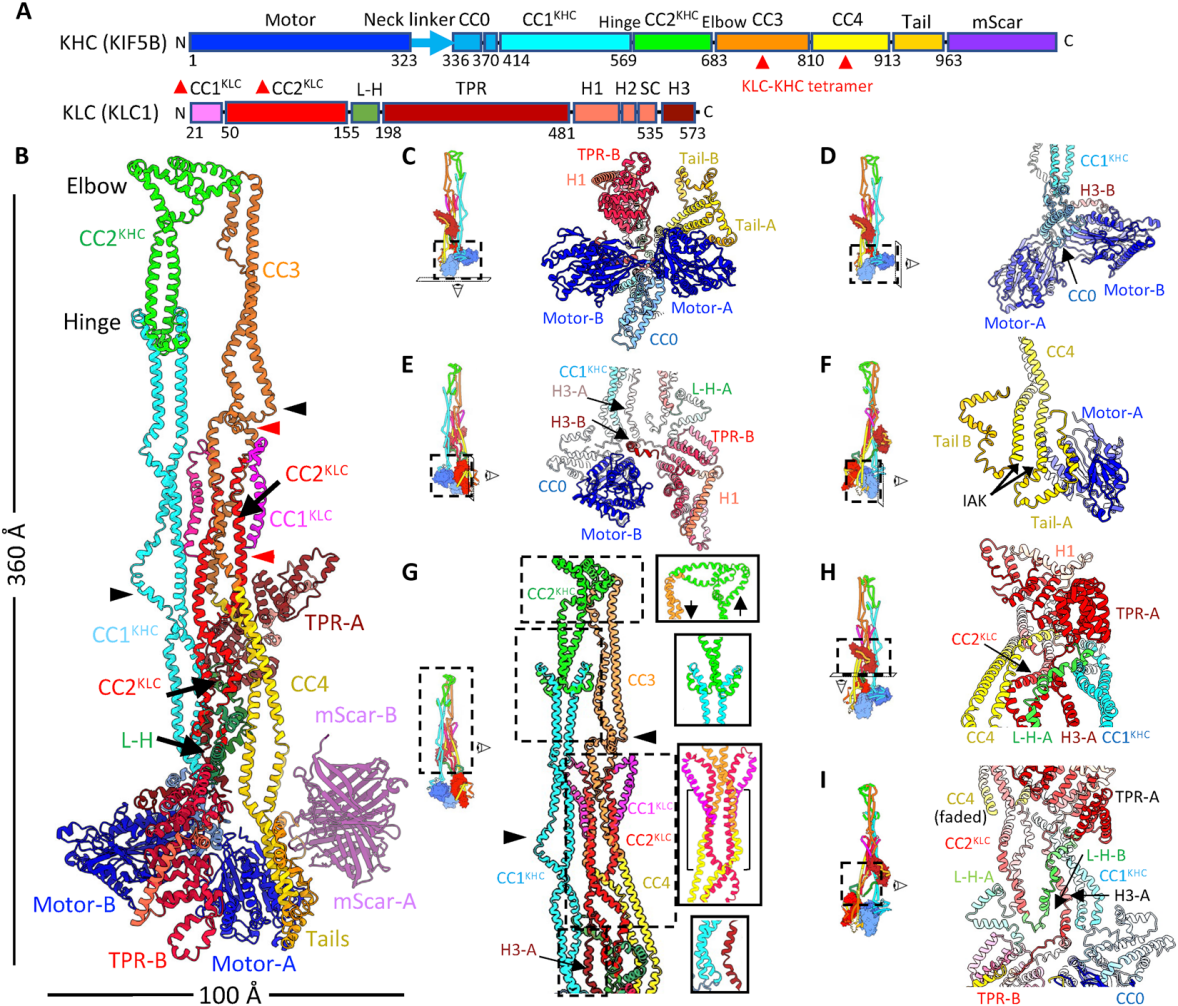
The organization of the KHC and KLC domains in the kinesin autoinhibited structure. (**A**) Domain maps of human KIF5B and KLC1. The KHC (KIF5B) domains include the motor domain, neck linker, CC0, CC1^KHC^, CC2^KHC^, CC3, CC4, and tails. The mScarlet (mScar) fused onto the C terminus is shown. The KLC (KLC1) includes CC1^KLC^, CC2^KLC^, L-H, TPR domain, and the H1, H2, SC, and H3 α-helical regions. Boundaries are indicated in residue numbers below, and each are colored to match colors for their models shown in (B) and (C). (**B**) The kinesin-1 KIF5B-KLC1 heterotetramer model with domains colored as shown in (A). KLC TPR-A and KLC TPR-B are shown in red and dark red, respectively. Black arrowheads refer to CC1^KHC^ and CC3 regions with supertwisted or untwisted coiled-coil regions, respectively. (**C**) Bottom view showing the KHC motor-A, motor-B, tail-A, tail-B, and the KLC TPR-B. (**D**) Side view showing the KHC dimeric motor-A and motor-B domains and the packed CC0 and CC1^KHC^ domains. (**E**) Side view showing the KHC motor-A interacting with KLC TPR-B and the self-folded state of CC1^KHC^ and CC4. (**F**) Side view showing the KHC motor-B interacting asymmetrically with tail-A and tail-B, which emerge from CC4. The IAK motifs, labeled with arrows, lie at the end of CC4. (**G**) Side view showing the coiled-coil regions of kinesin including CC1^KHC^, CC2^KHC^, CC3, CC4, CC1^KLC^, CC2^KLC^, and H3. The KHC-CC N and C termini are labeled. (**H**) Top tilted view showing the KLC TPR-A bound to the two KHC halves stabilizing CC1^KHC^ folding onto CC4. (**I**) Side view of the structure, with CC4 faded, showing the CC1^KLC^ and two directions of the L-H-A and L-H-B extending toward the KLC TPR-A and KLC TPR-B.

The KHC and KLC helical, coiled-coils, globular motor, and TPR domains generally matched their experimentally determined or AlphaFold-predicted structures (*22*, *23*, *26*) (fig. S9), but the structure revealed previously unknown intra- and intermolecular interactions that assemble the autoinhibited state, which are described below in detail. The dimeric KHC consists of N-terminal motor domains (motor-A and motor-B), followed by a series of dimeric coiled coil–containing segments: CC0, CC1^KHC^, CC2^KHC^, CC3, and CC4 (Figs. 1, D and E, and 2, A and B; and movie S2). The dimeric KLCs consist of two N-terminal KHC-binding KLC coiled-coil regions (CC1^KLC^ and CC2^KLC^), followed by linker-helical (L-H) regions (residues 164 to 198) and a cargo-binding TPR domain (residues 199 to 479). The structure reveals KLC regions C-terminal to the TPR domains; KLC C-terminal helical regions include helix-1 (H1; residues 480 to 509), helix-2 (H2; residues 510 to 522), SC (residues 522 to 535), and helix-3 (H3; residues 535 to 575). These KLC regions are all well-ordered α-helical regions and are crucial for maintaining the kinesin autoinhibited state (see below; Fig. 2 and movie S3).

### A structural model for autoinhibition of motor activity via extensive intramolecular self-folding

The overall 36-nm structure is composed of the KHC dimer folding back on itself, aided by the KLC domain interactions (Fig. 1, D and E). This lambda-shaped fold of the KHC comprises a “forward” arm (CC0, CC1^KHC^, and CC2^KHC^) and a “return” arm (CC3 and CC4), connected by a U-shaped structure, the elbow (*23*), which facilitates the 180° turnaround at the C terminus of CC2^KHC^ (Fig. 2, B to I). This antiparallel arrangement holds the N-terminal motor domains in an orientation incompatible with MT binding, providing a direct structural basis for autoinhibition of motor-domain interactions with MTs. The entire KHC assembly is further stabilized by the asymmetrically folded KLC dimer where TPR-A binds against the antiparallel KHC coiled-coil regions, while TPR-B bifurcates the motor domains in the head-to-tail KHC interaction interfaces (Figs. 1C and 2, B and C).

Several interactions lock the KHC motor domains into this inhibited conformation. The neck coiled coil (CC0) forms a dimeric four-helix bundle that is packed between the two motor domains, orienting them perpendicular to the particle’s long axis (Fig. 2, C to E). This state is reinforced by the KHC tails (tail-A and tail-B) (Fig. 2F), which emerge from the CC4 return arm to interact with only one motor domain (motor-A) (Fig. 2F). The clear observation of the dimeric mScarlet β barrels fused to the C termini of the KHC tails reinforces the order and the location of both KHC tails while binding a single motor domain (motor-A) (Fig. 2F). This asymmetric 2:1 tail-to-motor interaction is consistent with biochemical findings but is structurally distinct from contacts observed in prior models developed from isolated KHC fragments or KHC motor-tail fusion MT-bound complexes (*33*, *40*). Last, the previously designated “hinge” region (*31*, *41*) at the junction of CC1^KHC^ and CC2^KHC^ (Fig. 2G) is observed and is in good agreement with recent computational models and negative-stain EM data (*22*, *23*), validating another key feature of the KHC coiled-coil fold. Despite the lower resolution, the model building of the cryo-EM maps for CC1^KHC^ and CC4 coiled coils suggests that they adopt supertwisted or undertwisted coiled-coil conformations, respectively, when compared to their AlphaFold models [Fig. 2, B and G (black arrowheads), and fig. S5, B and C], suggesting the potential local relief of the molecular stress built-up in KHC coiled coil–containing regions upon self-folding; this is consistent with previous findings that kinesin-1 KHC changes coiled-coil propensity (*42*). Similar changes in coiled-coil twist are observed in the dynein stalk domain and the bicaudal D (BICD2) dynein activator (*43*, *44*).

The autoinhibited kinesin structure revealed an unexpectedly asymmetric organization of the dimeric KLC subunits (Fig. 2, E, H, and I). CC1^KLC^ folds around each KHC CC3-CC4 in antiparallel manner leading to CC2^KLC^, which runs parallel to CC3-CC4 (Fig. 2G). The N termini of the CC2^KLC^ helices form a tetrameric parallel coiled-coil bundle with KHC CC3-CC4, resulting in heterotetramer assembly (red arrowheads in Fig. 2, A, B, and G). This produces a parallel eight helical bundle in this region sandwiched between CC1^KHC^ and CC4 (Fig. 2, B and G). Following this tetrameric KHC-KLC assembly zone, the remaining CC2^KLC^ 90 residues assemble into homodimeric coiled coils that terminate with two L-H regions (L-H-A and L-H-B; Fig. 2, E and G to I). Although the L-H-A and the L-H-B regions emerge from the symmetric CC2^KLC^ dimer, they fold in opposite directions toward the asymmetrically positioned KLC-TPR domains (Fig. 2, B, E, H, and I). KLC TPR-A bridges alongside the two KHC-KLC coiled-coil arms (Figs. 1 and 2H), while KLC TPR-B is located in between the two motor domains and closely interacts with motor-B (Figs. 1 and 2B). The total buried surface area by KHC-KLC interaction is ~5500 Å and represents mostly coiled-coil and TPR interaction interfaces (fig. S8).

The autoinhibited kinesin cryo-EM structure is a substantial departure from previous models generated by AlphaFold or from negative-stain EM data (*22*) (fig. S9). Multiple AlphaFold models show the symmetry and extensive variability in the KLC-TPR domains binding to the coiled coils, and the mScarlets associated with KHC tails, which does not accurately predict our cryo-EM model (fig. S9B). Other key differences include the overall organization of the KHC coiled coils, the binding interfaces of the KLC-TPR domains, and the orientation of the motor domains. Notably, while the general position of the elbow is consistent with that reported by Tan *et al.* (*22*), its structure is fundamentally different. Instead of the sharp turn predicted by AlphaFold, our structure reveals a broad, U-shaped bend spanning ~40 residues (KIF5B; residues 640 to 686) that guides the KHC back upon itself (Fig. 2, B and G). Furthermore, our cryo-EM model provides the first complete structural view of the autoinhibited states of the neck coiled coil (CC0) (Fig. 2, C and D) and the C-terminal KHC tails (Fig. 2F), clarifying their crucial roles in kinesin autoinhibition and regulating activation.

### XL-MS validates the interfaces in kinesin autoinhibited structure

**Fig. 3.**
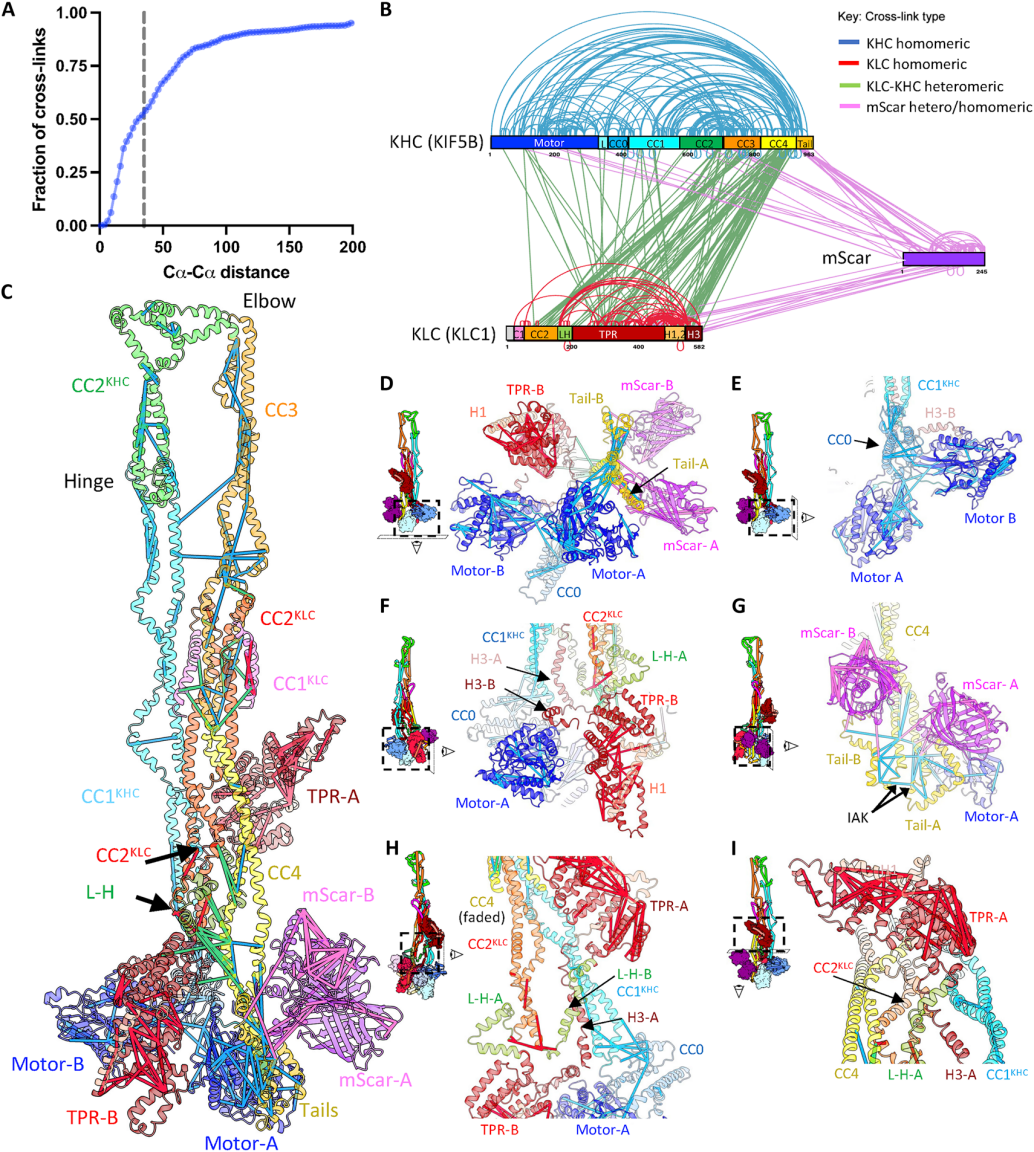
XL-MS validates the kinesin autoinhibited structure. (**A**) Cumulative distribution function plot showing the fraction of the 572 cross-links whose Cα-Cα distance is less than the indicated distance. The dashed line denotes the 35-Å cutoff. (**B**) A linear domain map of KIF5B (top left) with the fused mScarlet (center right) and KLC1 (bottom left) with internal cross-links highlighted in blue, pink, and red, respectively. The heteromeric cross-links between KIF5B and KLC1 are shown in green, and the heteromeric cross-links between mScarlet and KIF5B and KLC1 are shown in pink. (**C**) Cross-links identified in kinesin-1, illustrated in the context of the cryo-EM structural model of kinesin KIF5B-mScarlet-KLC1 heterotetramer. In total, 572 cross-links were successfully mapped onto the kinesin cryo-EM structure, of which 297 with Cα-Cα distances less than 35 Å are displayed. The view matches that in Fig. 2 (B and F). (**D**) Bottom view showing the KHC motor-A, motor-B, tail-A, tail-B, and the KLC TPR-B. The view matches that in Fig. 2C. (**E**) Side view showing the KHC dimeric motor-A and motor-B domains and the packed CC0 and CC1^KHC^ domains. The view matches that in Fig. 2D. (**F**) Side view showing the KHC motor-B interacting with KLC TPR-B and the self-folded state of CC1^KHC^ and CC4. The view matches that in Fig. 2E. (**G**) Side view showing the KHC motor-A interacting asymmetrically with tail-A and tail-B, which emerge from CC4. The view matches that in Fig. 2F. (**H**) Top tilted view showing the KLC TPR-A bound to the two KHC halves stabilizing CC1^KHC^ folding onto CC4. The view matches that in Fig. 2H. (**I**) Side view of the structure, with CC4 faded, showing the CC1^KLC^ and two directions of the L-H-A and L-H-B extending toward the KLC TPR-A and KLC TPR-B. The figure matches that in Fig. 2I.

To understand the multisubunit interactions within the autoinhibited kinesin in relation to the cryo-EM structure, we carried out XL-MS of the same autoinhibited kinesin heterotetramer used in our cryo-EM structures, but using BS3 cross-linker (see Materials and Methods). In total, we identified 592 unique cross-links in the kinesin sample, including 279 intra-KIF5B cross-links, 106 intra-KLC1 cross-links, 115 intersubunit cross-links between KIF5B and KLC1, and 92 cross-links involving mScarlet, although 20 cross-links were not mapped as they lie within the disordered KLC N terminus (Fig. 3C and fig. S10). Of these 572 cross-links that could be mapped onto the kinesin autoinhibited cryo-EM model, 297 (52%) satisfy the 35-Å distance cutoff (Fig. 3, A and C). Since our cryo-EM structure determination and flexibility analysis reveal flexible motions in various regions of the kinesin autoinhibited structure (movie S1), we also evaluated slightly longer cross-links (35 to 50 Å) and mapped those onto the structure in comparison to the shorter distance cross-links (fig. S11). With these additional cross-links, 68% of the cross-links are consistent with the cryo-EM structure (fig. S11A). These XL-MS data are consistent with the kinesin autoinhibited structure model, both in overall architecture (Fig. 3C and fig. S11, C to F) and in terms of specific interdomain contacts (Fig. 3, D to I, and fig. S11, C to F).

Close-up views of the cross-links reveal numerous short-range cross-links within both folded KHC and KLC domains, showing a strong consistency with the domain contacts resolved in our cryo-EM structure (Fig. 3, C to I). For example, 48 cross-links were identified within the KHC motor domains; two and six cross-links were observed connecting the KHC motor domain to CC0 and CC4 tail, respectively (Fig. 3, C to E). In addition, we noticed two short-range (<20 Å) cross-links connecting CC1^KLC^ and CC3 domains (Fig. 3C). All these observations match the antiparallel fold back of the KHC coiled coils. Fourteen cross-links were observed between the KHC CC3-CC4 and CC2^KLC^, which confirms the dimerization of the KHCs and KLCs to form a tetramer bundle (Fig. 3C). We also identified three short-range cross-links connecting the KLC L-H and TPR region to the KHC CC4 region (Fig. 3C). Together with the observation of numerous long-distance cross-links between the KLC-TPR domain region and nearly all regions of KHC, these findings are consistent with our asymmetric model for the two TPR regions within the kinesin heterotetramer (Fig. 3C). We observe extensive cross-links within each mScarlet domain and between the mScarlet domains and KHC motor, CC0, CC4, and tail domains (Fig. 3, C, D, and G). Extended distance cross-links (35 to 50 Å) mostly map to interfaces between the KLC TPR-B and KHC tail domains and map to the interface between KLC TPR-A and the CC1^KHC^ and CC4 and CC2^KLC^ (fig. S11, D to F). This suggests that the KHC tail and coiled-coil interfaces with the KLC-TPR domain may exhibit flexible motions, consistent with flexibility analyses (movie S1).

We also compared our XL-MS data to previously published KIF5B-KLC1 kinesin heterotetramer XL-MS dataset by Tan *et al.* (*22*) (fig. S10E). Mapping their identified cross-links onto our cryo-EM model revealed that 147 of 233 cross-links (63%) fall within the 35-Å distance cutoff, indicating a high level of consistency with the model (fig. S10, B and E). Notably, their dataset did not include the KIF5B-fused mScarlet. Our XL-MS data provide confidence in the assigned location of mScarlet by showing five short-range cross-links between the mScarlet and KHC CC4-tail domains (Fig. 3, C, D, and G). In addition, we detected numerous long-range cross-links linking mScarlet to other domains, including the KHC motor, coiled-coil, and tail regions, as well as the KLC-TPR domain and helical CC2^KLC^ and L-H regions (Fig. 3C and fig. S11). Consistent with this model, XL-MS cross-links show the Lys^922^ in IAK motif cross-links to residues in KHC motor (residues 166 and 213) and cross-links to residues in the KHC tail (residues 903, 908, and 933), which lie in other helices of the W-hairpin (Fig. 3G). Hence, our XL-MS data and that of Tan *et al.* (*22*) serve as orthogonal validation of the cryo-EM structural model, including the location of the KIF5B-fused mScarlets and the location of the KHC tails that were previously improperly placed in the negative-stain EM or AlphaFold models (*22*) (fig. S9).

### Dimeric KHC C-terminal tails bind to one motor domain in the autoinhibited state

The KHC C-terminal tails, which are crucial for forming the autoinhibited state (*22*, *23*, *45*), are revealed to be ordered in the structure as W-shaped helical hairpin domains, and their positions were validated by the β barrel–shaped mScar-A and mScar-B fused onto their C termini (Fig. 2, B, C, and F). Each KHC tail forms a helical W-hairpin emerging as dimers at the end of CC4. Similarly, W-shaped hairpin motifs are found in many other proteins with two-stranded antiparallel coiled coils and are crucial for binding other proteins or RNA (*46*). The dimeric KHC tails interact with the motor domains in a notably asymmetric fashion. Only one tail (tail-A) directly engages a single motor domain (motor-A), binding near the α2 (P-loop) and α3 (switch II) helices. Our model suggests that the conserved IAK motifs are positioned at the junction of CC4 and the first tail helix (Fig. 2, C and F). The KHC tails also make contacts with the KLC TPR-B on the other side, as evidenced by XL-MS cross-links (figs. S10 and S11). This new KHC tail-motor binding site is consistent with differential adenosine 5′-diphosphate (ADP) to adenosine 5′-triphosphate (ATP) nucleotide exchange between the two motor domains (*47*). The IAK motif junction regulates dimeric KHC tail orientation to motor-A. The second tail (tail-B) remains unbound from motor-A but may shield an auxiliary MT interaction site at the end of CC4, which is consistent with finding that KHC tails lower the KHC CC4 affinity for MTs (*48*). This asymmetry in KHC tail-to-motor interactions are further evidenced by the staggered positions of the C-terminally fused mScar-A and mScar-B; mScar-A fused to the bound tail-A is positioned ~10 Å lower than the mScar-B fused to the unbound tail-B, suggesting that binding to motor-A constrains and tightens the tail-A structure (Figs. 1 and 2).

This model of the KHC tail-motor interaction differs significantly from previous structures derived from cocrystals of isolated motor domains and tail peptides or from cryo-EM maps of MT-bound motor-tail fusion constructs (*33*, *40*). Our autoinhibited kinesin cryo-EM structure demonstrates that the native inhibitory KHC tail-motor domain contacts are critically dependent on the structural context—specifically, antiparallel orientation of the dimeric KHC CC4 tails to one motor domain (motor-A) and to a KLC-TPR domain (TPR-B) (Fig. 2, B and C). Structural comparisons of MT bound the isolated motor-tail domains model to a crystal structure of these domains show that if motor-A aligned between the two models, then motor-B is found in two different conformations while only binding to the tail peptide between the two motor domains (fig. S12, A and B). In those structures, the KHC tail peptide binds near β-4 of both isolated tail-motor domain structures. This differs from our cryo-EM structural model showing one tail binding near α2 and α3 of motor-A in the autoinhibited kinesin structure (fig. S12C). The new tail binding site revealed by our cryo-EM structure lies on the opposite side of the motor, next to its P-loop (fig. S12C). The KLC-TPR domain and KHC-CC0 interactions with motor-A and motor-B occlude the space for any of the states in which motor-B binds the KHC tail, as observed in the isolated crystal structure or MT-decorated cryo-EM maps of those constructs (Fig. 2G). Furthermore, our structural model supports that the KHC C-terminal tail-to-motor domain interactions in addition to the CC1^KHC^ and CC3-CC4 are likely preserved even in the absence of KLCs, supporting previous low-resolution negative-stain EM studies on KHC dimers (*22*, *24*), highlighting the tail-to-motor interaction as a fundamental feature of KHC autoinhibition and a crucial role of the KLCs in enhancing kinesin autoinhibition.

### Asymmetric binding of the KLC-TPR domains to either a KHC coiled coil or a KHC motor domain

Despite recent insights into autoinhibited kinesin (*22*, *23*), the precise positioning of the KLC-TPR domains has remained an open question. Our cryo-EM structure resolves this by revealing that the two KLC-TPR domains bind in an asymmetric manner to the symmetric KHC dimer (Figs. 1, D and E; 2, B, C, E, and H; and 4A). Instead of engaging equivalent sites, each TPR domain binds to a distinct region of the KHC, adopting unique roles. One TPR domain (TPR-A) bridges across the CC1^KHC^, CC3-CC4, and CC2^KLC^ antiparallel coiled-coil bundle (Fig. 2H), while the other (TPR-B) separates the KHC motor-A and motor-B domains, possibly constraining their movement (Fig. 2, B, C, and E).

**Fig. 4.**
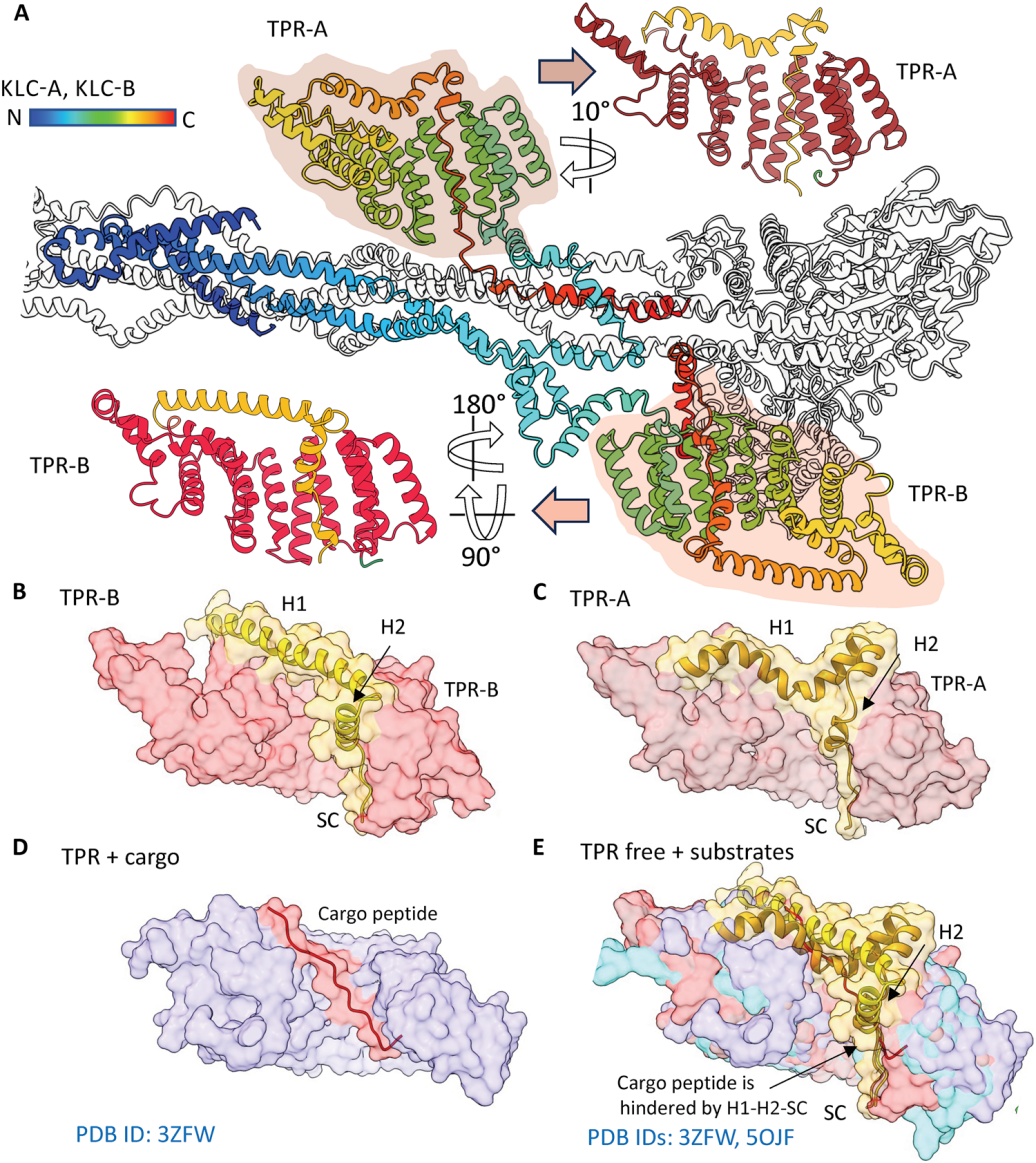
The topology and self-regulation of the KLC subunits in lambda kinesin autoinhibited structure. (**A**) A general view of the kinesin structure with KHC colorless and KLC is shown in rainbow gradient from the N to C terminus. Each TPR domain is shown and its orientation transformed via arrows to orient them in a comparable manner. The isolated KLC-TPR domain models show the role of the KLC C-terminal helical regions, colored in gold, autoinhibiting access to the cargo-binding concave surface of the TPR domains. (**B**) A view of the KLC TPR-B domain with the helical regions of the TPR fold shown in surface representation with the KLC C-terminal helical regions shown in ribbon form. (**C**) A view of the KLC TPR-A domain in the same orientation described in (B). (**D**) A view of the KLC-TPR domain structure [Protein Data Bank (PDB) ID: 32FW] with the Y-acidic motif cargo peptide bound to the concave surface of the KLC-TPR domain (*26*). (**E**) An overlay of the KLC-TPR domain structure (brown and salmon) in two states described here and the free (cyan; PDB ID: 5OJF) and Y-acidic motif cargo-peptide bound form (blue; PDB ID: 3ZFW) overlaid. The SC and H2 autoinhibit the TPR domain and sterically hinder cargo peptide binding.

TPR-A stabilizes the folded KHC architecture by bridging the CC1^KHC^-CC2^KHC^ and CC3-CC4 arms (Fig. 2H). It uses its narrow surface, composed of interhelical repeat turns, to bind the KHC coiled coils, maintaining them together in their folded conformation (Figs. 1 and 2). This interface is constrained by the TPR-A N and C termini bound to KHC coiled coils (see below; Fig. 2, B and I). This interaction is critical for maintaining the overall integrity of the lambda particle. This region is a major source of flexibility around these coiled coils (movie S1). In contrast, TPR-B is positioned in between motor-A and motor-B domains, on the opposite side of the dimerized KHC neck or CC0. The positioning of TPR-B between the motor domains may explain the elevated autoinhibition of kinesin KHC in the presence of KLC (*32*, *49*) and is consistent with the report of motor domain bifurcation by KLC-TPR domain (*21*). TPR-B interacts closely with motor-B, using the helices of its TPR repeats 2 to 5 to lock motor-B in a restricted state (Fig. 2, C and E). The amino acid sequences at these unique KLC-KHC interaction interfaces are highly conserved across species (fig. S13), underscoring their functional importance.

Beyond constraining the KHC head-to-tail fold in the autoinhibited state, our cryo-EM structure suggests another mechanism for KLC autoinhibition (Fig. 4). In both KLC subunits, ordered C-terminal helical regions—H1, H2, and an SC motif—fold back to occupy the concave, cargo-binding face of their respective TPR domains, sterically preventing adapter protein engagement (Fig. 4, B to E). This complex, asymmetric arrangement is accommodated by distinct conformations of the KLC N- and C-terminal linker helices (L-H and H3) (Fig. 2, H and I). For example, the linker following TPR-A (H3-A) binds along CC1^KHC^, while the linker following TPR-B (H3-B) binds across the top of motor-B (Fig. 2, E and I). Our work provides the first complete view of all KLC helical segments within the context of the fully autoinhibited kinesin structure.

### The KLC-TPR domains are autoinhibited by C-terminal helical sequences

Previous studies proposed that KLC is autoinhibited via an N-terminal “LFP” sequence that competes with cargo proteins for binding to the TPR domain (Fig. 4D) (*50*, *51*). We find that the concave cargo-binding groove of each TPR domain is occupied by a series of helices (H1, H2, and SC motif) that are C-terminal to the TPR domain itself (Figs. 2 and 4, A to C). H1 acts as a connector that folds back from the TPR domain, positioning H2 and the SC motif to sterically block the interface for Y- or W-acidic cargo motifs (Fig. 4, B to D). This self-inhibited state is further locked in place by the most C-terminal helix, H3, which anchors the KLC to adjacent KHC domains (CC1^KHC^ or motor-B). A subtle difference exists between the two KLC-TPR domains: H1-A is more bent toward the concave face of TPR-A, while H1-B adopts a more extended conformation along TPR-B (Fig. 4, B and C). Our structure confirms that the KLC-TPR domains exist in an autoinhibited state but reveals a fundamentally different mechanism for cargo inhibition than previously suggested (*50*, *51*), driven by the KLC C-terminal helical elements (Fig. 4E).

Our findings directly contrast with a previously proposed N-terminal LFP mechanism (*50*). We attribute this discrepancy to the different experimental contexts: Our structure represents the full-length kinesin heterotetramer, whereas earlier studies used isolated KLC proteins with truncated C termini that lacked the inhibitory helices we observe here (*50*). The context of the full kinesin complex is therefore essential for revealing the mechanism of KLC autoinhibition.

### Restrictions of the kinesin motor domains in the autoinhibited structure

**Fig. 5.**
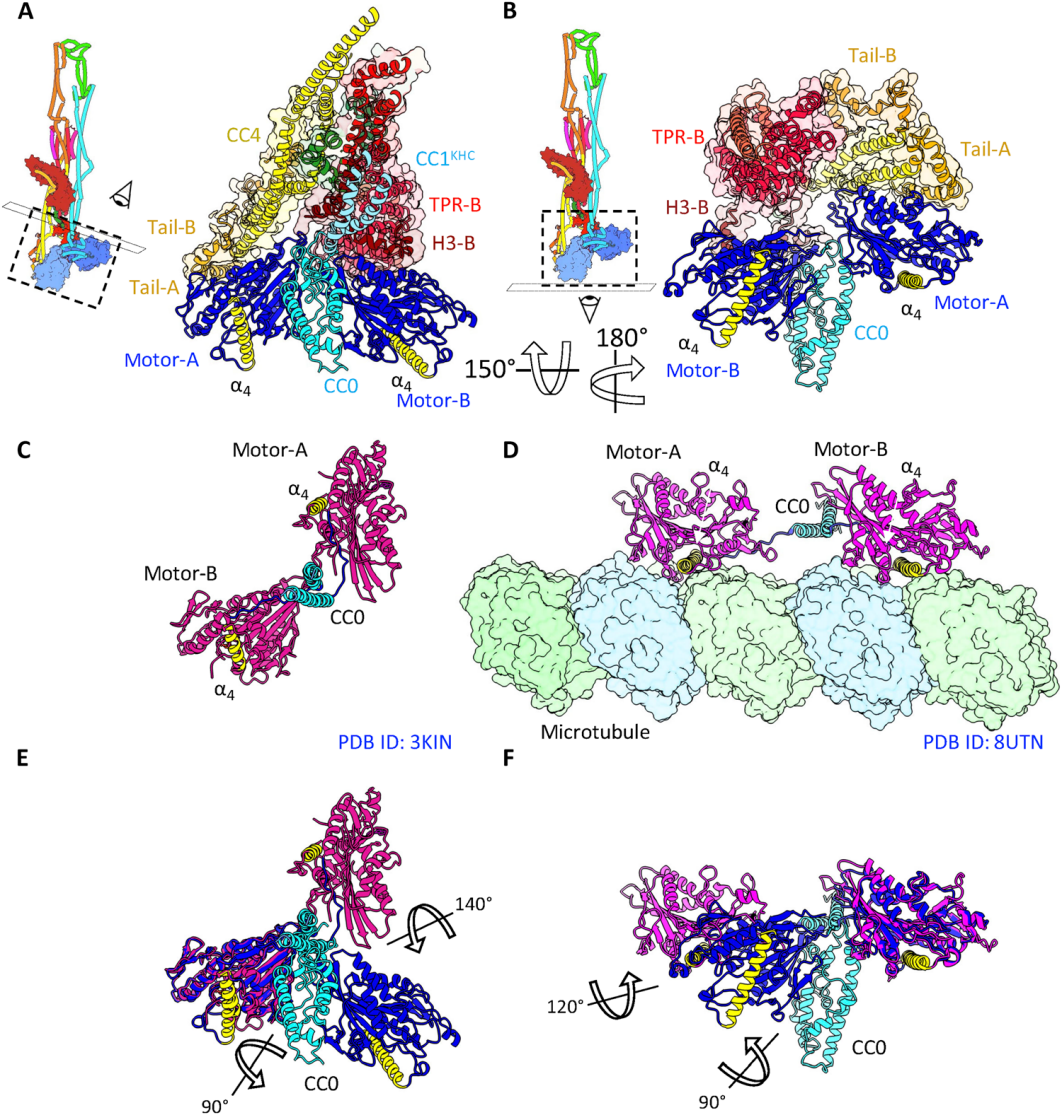
The kinesin motor domains are restricted from motility by the lambda autoinhibited kinesin structure interactions. (**A** and **B**) Two views of the kinesin structure showing the KHC motor-A and motor-B (blue) bound by the KHC tail-A and tail-B (orange and gold) and the KLC-TPR domain (red) and CC0 (cyan) packed beneath CC1^KHC^. The MT-binding site of each motor domain is highlighted with α4 helix shown in yellow. (**C**) A view matching the orientation shown in (A) of the dimeric kinesin motor domain crystal structure (*52*) (PDB ID: 3KIN) showing the motor domains (magenta) and the CC0 (cyan). The MT-binding site of the motor domain MT is highlighted with α_4_ helix shown in yellow. (**D**) A view matching the orientation shown in (A) of the dimeric KIF1A motor domain cryo-EM model along the MT protofilament (*53*) (PDB ID: 8UTN) showing the motor domains (magenta) and the CC0 (cyan). The MT-binding site of the motor domain is engaged with the MT, and this binding site is highlighted with α4 helix shown in yellow. (**E**) Overlay of view of the structures shown in (A) and (C) was aligned via motor-B, with the structural transitions of CC0, and motor-A domains shown in (A) with those shown in (C). (**F**) Overlay of view of the structures shown in (B) and (D), aligned via motor-B, with the structural transitions of CC0, and motor-A domains shown in (B) with those shown in (D).

While it is known that full-length kinesin motility is strongly repressed (*32*, *41*), the precise structural mechanism has been unclear. Our structure reveals that the dimeric KHC motor domains are locked into a tightly constrained, nonprocessive MT-binding configuration through a multipoint locking mechanism (Fig. 5, A and B). This is achieved by specific interactions with one KHC C-terminal tail (tail-A), a KLC-TPR domain (TPR-B), and the neck coiled-coil (CC0): Tail-A binds directly to motor-A, while TPR-B separates the two motor domains and tightly engages motor-B. Simultaneously, the KHC CC0 dimer is packed into a unique conformation against the two motor domains at their dimerization junction and further restricts their flexibility from opposite side to the TPR-B and KHC tail binding sites (Figs. 2, B to F, and 5, A and B).

To understand the functional consequences of this locked arrangement, we compared it to motile or unbound kinesin structures, including the MT-bound KIF1A dimer and the free rat kinesin motor dimer crystal structure (*52*, *53*). We aligned the autoinhibited kinesin structure to the KIF1A MT-bound cryo-EM structure and the free kinesin-1 motor dimer crystal structure via motor-B domain (Fig. 5, C to F). This comparison reveals that, when structures are aligned by the motor-B domain, a marked reorientation is observed in the other motor domain. Motor-A of our autoinhibited kinesin structure is twisted by 120° to 140° around its neck linker junction relative to its position in the processive motile or free states (Fig. 5, C to F). As a direct result, the MT-binding interfaces, denoted by α4 helices, of the two motor domains are severely misaligned and cannot engage the MT lattice sites simultaneously (Fig. 5, E and F). Therefore, our structure demonstrates that kinesin autoinhibition physically prevents its processive motility by locking the motor domains in a conformation where their free processive stepping mechanism is impossible. In addition, our flexibility analyses (movie S1) and classifications (figs. S2, bottom, and S3) suggest that motor-B that does not interact with tail-A or tail-B is likely to be more flexible, which suggests that it may be the motor domain able to initiate MT binding in the autoinhibited state.

### MAP7D3^CT^ binding to CC1^KHC^ destabilizes the kinesin autoinhibited state

MAP7 family proteins are critical for kinesin function in vivo (*34*, *36*). While it is established that MAP7 enhances kinesin’s MT association, its direct role in activating motility has been ambiguous (*34*–*36*). Some studies suggest full activation requires a cargo-adapter protein (*32*), while others observed increased motility that could be attributed to a higher MT landing rate rather than true activation (*34*, *35*). More recently, the paralog MAP7D3, which binds kinesin more tightly, was shown to enhance motility even in the truncated K560 constructs (KIF5B; residues 1 to 560), composed only of the motor domains, CC0, and CC1^KHC^, suggesting a more direct recruitment mechanism (*34*).

**Fig. 6.**
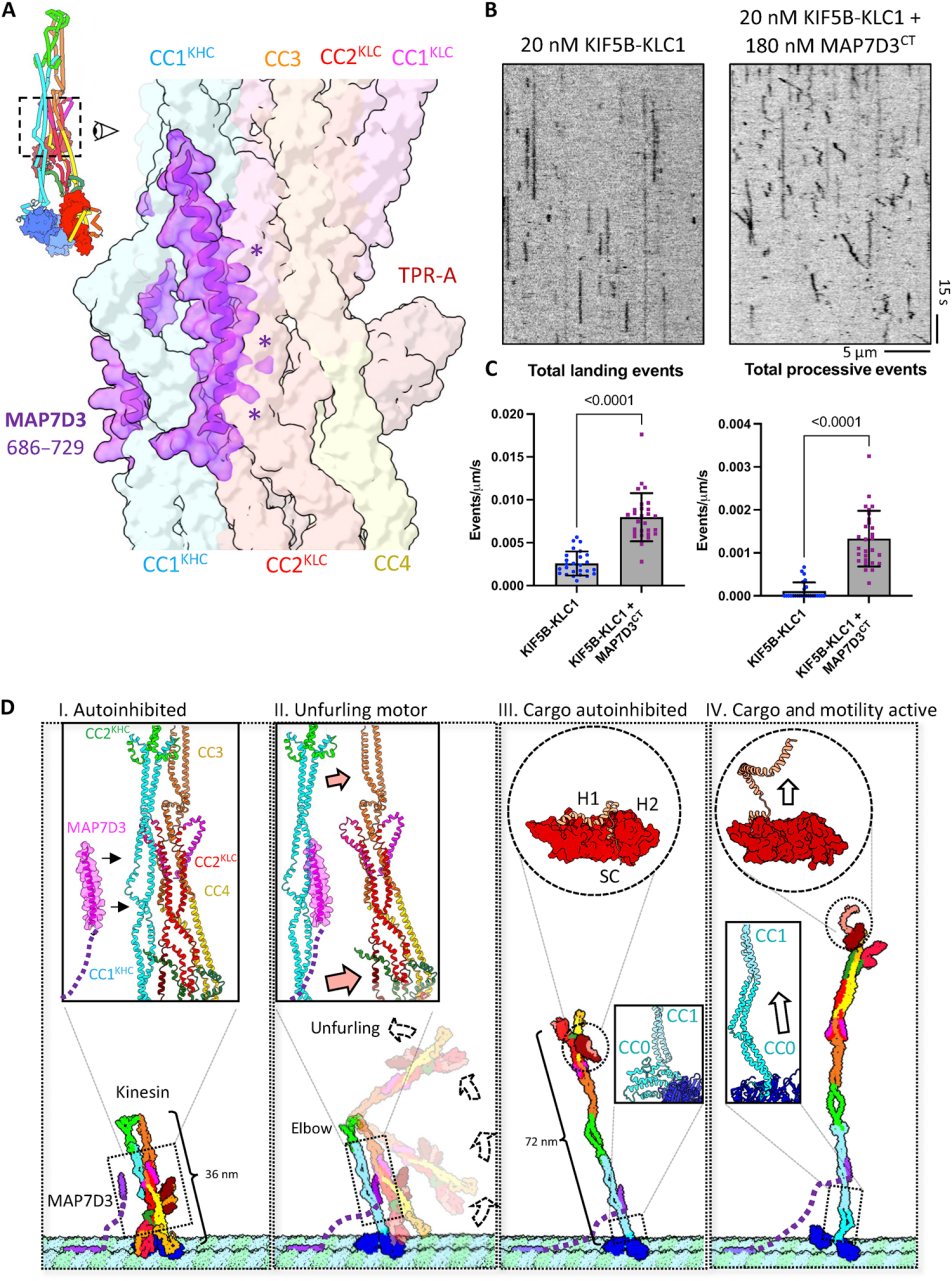
MAP7D3^CT^ is predicted to bind CC1^KHC^, sterically competing with the kinesin autoinhibition interfaces, increasing MT landing rate and enabling kinesin motility along MTs. (**A**) An overlay of the AlphaFold3 model of MAP7D3^CT^ (residues 686 to 728) (purple envelope and ribbon) binding to CC1^KHC^ onto the CC1^KLC^-CC2^KLC^-CC4 kinesin autoinhibited structure (faded surface colors), revealing that MAP7D3^CT^ binding induces steric hindrance at the CC2^KLC^ and CC4 interface on CC1^KHC^. Details are shown in figs. S10 and S14. (**B**) Kymographs of kinesin reconstituted with MTs by TIRF microscopy. Left: Autoinhibited kinesin undergoes transient and static association events along MTs. Right: In the presence of MAP7D3^CT^, kinesin shows enhanced landing rates and activation of processive motility toward MT plus-ends (left end). (**C**) Left: Quantitation of MT landing events for kinesin in the absence and presence of MAP7D3^CT^. Right: Quantitation of processive motility events. Student’s *t* test was used to compare datasets; *P* values are provided. (**D**) A multilevel model illustrating MAP7D3-driven kinesin activation through disruption of the autoinhibited lambda conformation, shown in four stages (I to IV). In each state, the bottom panel shows the overall kinesin conformation with domains colored as in Fig. 2; top panels show magnified close-up views of specific regions undergoing structural change. In state I, MT-bound MAP7D3 engages the MT lattice, and its activation domain binds CC1^KHC^, causing a steric clash with CC3-CC4 and CC2^KLC^. In state II, MAP7D3 binding destabilizes the autoinhibited complex, triggering a large-scale rearrangement that sequentially releases the motor domains. In states III and IV, the unfurled kinesin undergoes unraveling of the neck coiled-coil (CC0) (central panels), while the KLC-TPR domain cargo-binding site is unmasked as the C-terminal inhibitory helices are displaced from the TPR groove (top). Full unraveling of CC0 and relief of TPR autoinhibition together release kinesin to bind cargo and undergo processive motility.

To investigate how MAP7D3 activates kinesin, we first used AlphaFold3 to model the interaction between the MAP7D3 C-terminal domain (MAP7D3^CT^; residues 476 to 876) and the KHC dimer (*31*, *54*, *55*). The AlphaFold3 model predicted that residues 680 to 729 of MAP7D3 form a four-helix bundle with the CC1^KHC^ region, an interaction that would sterically clash with the autoinhibited fold of kinesin and disrupt critical stabilizing contacts (Fig. 6A and fig. S13). To test this hypothesis, we performed single-molecule total internal reflection fluorescence (TIRF) microscopy assays with kinesin heterotetramer (KIF5B-mScarlet KLC1) with and without MAP7D3 to assess whether MT binding is necessary for MAP7D3^CT^ interactions. As expected, the kinesin heterotetramer was strongly autoinhibited in the absence of MAP7D3^CT^ and bound statically along MTs (*32*, *34*–*36*). In stark contrast, the addition of MAP7D3^CT^ triggered a ~12-fold increase in the number of processive motility runs, which showed a higher motility velocity than the paused kinesin without MAP7D3 (Fig. 6, B and C). These results demonstrate that MAP7D3^CT^ can relieve kinesin autoinhibition, consistent with the idea that MAP7D3^CT^-binding CC1^KHC^ disassembles the autoinhibited state, providing direct functional validation for our structural model.

This activation mechanism also explains how MAP7D3 enhances cargo binding, as shown in our previous work (*38*). Our model suggests that this occurs because disrupting the autoinhibited complex by interacting with CC1^KHC^ and thus liberates the KLC-TPR domains from CC1^KHC^, which is consistent with recent finding from Shukla *et al.* (*56*). The autoinhibited kinesin interacts with the MT and encounters MAP7D3^CT^ leading to the relief of autoinhibition to release kinesin to undergo processive motility runs. Therefore, MT-bound MAP7D3 functions as a dual-action activator and recruiter to the MT lattice. MAP7D3 sterically disrupts the kinesin autoinhibitory interface to directly trigger motility, which, in turn, unmasks the KLC-TPR domains to promote the recruitment of cargo adapters (*38*).

## DISCUSSION

Kinesin-1 is the founding and most extensively studied member of the kinesin superfamily. For decades, research on kinesin-1 has defined its processive motility mechanism (*3*, *18*, *57*), yet the molecular basis of its spatiotemporal regulation has remained poorly understood. It is well established that the kinesin heterotetramer is autoinhibited via intramolecular interactions (*41*, *45*, *48*), but a complete structural picture of this state has been missing and somewhat contradictory (*21*, *31*, *32*, *41*, *58*). Deletion of the KHC C-terminal tail region or newly identified KHC elbow regions leads to activation of enhanced kinesin processive motility (*23*, *41*). Recent work has converged on a model of sequential activation, in which factors such as MAP7 first activate motility, which, in turn, permits the loading of cargo via the KLC-TPR domains (*32*, *34*–*36*, *38*). However, the structural basis for this coordinated process—how the motor is inhibited from both moving and binding cargo and how activators could reverse this—has been a central and unresolved question.

Our cryo-EM structure and XL-MS analysis of the fully autoinhibited kinesin provide a structural basis of its autoregulation. We show that the complex is a precisely organized assembly in which long-range interactions create a dual-locked state. First, motor activity is suppressed by a network of asymmetric contacts in the autoinhibited state: The two KLC-TPR domains either bind together to the antiparallel folded CC1^KHC^ and CC4/CC2^KLC^ or separate the dimeric KHC motor-A and motor-B domains (Figs. 1 and 2 and movies S2 and S3). Flexibility analyses (movie S1) and the XL-MS studies (Fig. 3 and fig. S9) support these cryo-EM structural TPR interactions but suggest that they are dynamic. In conjunction with the KHC C-terminal tails, the KLC-TPR domains restrict the free motility of the KHC motor domains, which is essential for processive motility (Figs. 1, 2, and 5). Second, and unexpectedly, cargo binding by the KLC-TPR domain is independently blocked by the C-terminal KLC helical segments, in which H1, H2, and SC occupy the concave TPR cargo-binding groove, while H3 binds distant elements such as CC1^KHC^ that are in close proximity to maintain the autoinhibited state (Figs. 2 and 4). This mechanism of cargo peptide occlusion is distinct from previously proposed models (Fig. 4) (*26*, *51*). This dual-inhibited kinesin architecture provides a comprehensive blueprint fail safe for how kinesin robustly maintains an “off” state, while also revealing distinct structural points of regulation that can be targeted by activators to sequentially unlock its motility and cargo loading. It also provides a map to interpret the functional variation of the paralogs of KHC and KLC with distinct sequences at their C termini.

### A structure-based model for kinesin activation and the sequential loading of cargo

Our structural and biochemical data converge on a comprehensive model for the sequential activation of kinesin, integrating MAP7-mediated recruitment with the relief of the autoinhibition of both motility and cargo binding (Fig. 6). A previous study demonstrated that MAP7D3^CT^’s high affinity for kinesin-1, mediated through binding CC1^KHC^, enhances recruitment of the truncated, constitutively active KIF5B K560 motor fragment to MTs by increasing landing rates and promoting processive run length (*34*). Here, we extend these findings to the autoinhibited, full-length kinesin-1 heterotetramer and show that MAP7D3^CT^ similarly activates processive motility runs and enhances MT landing rate (Fig. 6). We propose a structural transition cascade that begins when MT-bound MAP7D3^CT^ recruits an autoinhibited kinesin heterotetramer, increasing its local concentration on the MT (Fig. 6, D and I). Consistent with previous reports (*34*, *35*) and our AlphaFold models (Fig. 6A and fig. S14), the MAP7D3^CT^ likely binds to CC1^KHC^. The high binding affinity of MAP7D3^CT^ to CC1^KHC^ sterically clashes with the CC3-CC4 and CC2^KLC^, destabilizing the interfaces between the two folded halves of the KHC/KLC coiled-coil interfaces (Fig. 6D, top insert of I and II). This initial disruption triggers a large-scale conformational rearrangement, leading to the unfurling of the full kinesin heterotetramer, nearly doubling its length from ~36 to ~72 nm, which releases inhibitory constraints on the motor domains, including the unraveling of the packed neck (CC0) coiled coil (Fig. 6D, III and IV, middle) to permit processive motility to the MT plus-end. Concurrently, this global unfurling liberates the KLC-TPR domains via the release of KLC-H3, causing the KLC C-terminal inhibitory helices H1, H2, and SC to dissociate from the TPR cargo-binding groove. This final step unmasks the cargo-binding sites of the KLC-TPR domain, rendering the now-motile kinesin fully competent for cargo-adapter engagement (Fig. 6D, III and IV, top). The unraveling of the packed-up state of the KHC neck (CC0) coiled coil due to absence of the autoinhibition interactions with the KLC-TPR and KHC tail domains leads to the free release of the KHC motor domains to undergo processive motility (Fig. 6D, III and IV, middle).

In conclusion, elucidating the intramolecular interactions that govern kinesin-1 autoinhibition has been a long-standing challenge in the cytoskeletal motor field. Our work provides a structural answer, revealing a comprehensive blueprint for how motor activity and cargo engagement are coregulated. By demonstrating how a network of asymmetric, long-range interactions creates a dual-locked state, this model establishes a clear foundation for future mutational studies to dissect these mechanisms both in vitro and in vivo. Furthermore, it provides a powerful framework for exploring functional differences in autoregulation across the entire kinesin superfamily.

## MATERIALS AND METHODS

### Protein expression and purification kinesin-1 heterotetramer and MAP7D3^CT^

For kinesin-1 expression, KIF5B-mScarlet and KLC1 were cloned into pACEBac1/pIDS vectors, and DH10MultiBac (Geneva Biotech) vector was transformed as recombined plasmid to generate bacmid, as previously described (*32*). Baculovirus was prepared by bacmid transfection using Cellfectin II reagent (Thermo Fisher Scientific), followed by 2 cycles of amplification leading to P2 virus. For protein expression, 400 ml of Sf9 cells (2 × 10^6^ to 3 × 10^6^ cells/ml) were infected with 4 ml of P2 virus and cultured for 65 hours at 27°C. Cells were harvested and resuspended in 25 ml of lysis buffer [50 mM Hepes-KOH (pH 7.5), 150 mM KCH_3_COO, 2 mM MgSO_4_, 1 mM EGTA, and 10% glycerol] along with 1 mM dithiothreitol (DTT), 1 mM phenylmethylsulfonyl fluoride (PMSF), 0.1 mM ATP, and 0.5% Triton X-100. After incubating on ice for 10 min, the lysates were centrifuged at 15,000*g* for 20 min at 4°C. The resulting supernatant was subject to affinity chromatography in which the supernatants were pumped over a column of Streptactin XT resin (IBA) for 1 hour at 4°C. The columns were then washed with excess amount of lysis buffer to remove unbound material, and the proteins were eluted in lysis buffer containing 100 mM d-biotin. Eluted proteins were further purified using size exclusion chromatography using TSKgel—a Phenomenex BioSep 5-mm SEC-s4000 500-Å column with a size of 600 mm by 7.8 mm—as previously described (*32*).

For MAP7D3^CT^ expression, MAP7D3^CT^ was cloned into bacterial expression vector and transformed into BL21-CodonPlus (DE3)–RIPL *Escherichia coli* (Agilent, Santa Clara, CA). Cultures of BL21-expressing MAP7D3^CT^ were grown at 37°C in Luria broth with kanamycin (50 μg/ml) until an optical density at 600 nm of 0.6. Protein expression was induced with 0.2 mM isopropyl-β-d-thiogalactopyranoside at 18°C overnight. Bacterial cultures were centrifuged at 5000*g*, and pellets were frozen. Bacterial pellets were thawed on ice and resuspended in purification buffer [PB; 50 mM tris-HCl (pH 8.0), 150 mM KCH_3_COO, 2 mM MgSO_4_, 1 mM EGTA, and 5% glycerol] freshly supplemented with 1 mM PMSF, 0.1 mM ATP, NucA nuclease, and protease inhibitor mix (Promega, Madison, WI). For purifying MAP7D3^CT^, 1 mM DTT was also added during this step. Bacteria were lysed by passage through an Emulsiflex C3 high-pressure homogenizer (Avestin, Ottawa, ON, Canada), followed by addition of 1% Triton X-100 for 5 min on ice. Lysed cells were then centrifuged at 22,769*g* for 20 min at 4°C. For affinity purification, the clarified lysates were incubated with resin as follows. MAP7D3^CT^ was incubated with Streptactin XT resin (IBA Lifesciences, Göttingen, Germany) for 1 hour at 4°C and washed with PB. MAP7D3^CT^ was further purified using a HiTrap Capto S cation exchange chromatography column (Cytiva) equilibrated in HB buffer [35 mM Pipes-KOH (pH 7.2), 1 mM MgSO_4_, 0.2 mM EGTA, and 0.1 mM EDTA (pH 7.1)]. Bound proteins were eluted with a 45-ml linear gradient of 0 to 1 M KCl in the HB buffer.

### Cryo-EM sample preparation and data collection

KIF5B-mScarlet-KLC1 at 1 mg/ml was analyzed on sucrose density gradient (fig. S1A). We then tested cross-linking with 0.01 to 0.08% (v/v) glutaraldehyde at room temperature for 30 min and analyzed on SDS–polyacrylamide gel electrophoresis (PAGE) gel to find optimal cross-linking concentration (fig. S1B). The sample was prepared for cryo-EM sample with 0.06% (v/v) glutaraldehyde with GraFix (*59*) system using 20 mM Hepes (pH 7.2), 100 mM KCl, 1 mM MgCl_2_, 5 mM β-mercaptoethanol (B-ME), and 1 mM EGTA, loaded onto 10 to 40% (v/v) sucrose gradient with KIF5B-mScarlet KLC1 (1 mg/ml), and ran for 16 hours at 4°C at 40000 rpm (fig. S1A). Sample was manually collected with a pump at 10-s interval of ~200 μl. Fractions containing KIF5B-mScarlet KLC1 was then neutralized with 1 mM tris-HCl (pH 7). Cross-linked samples were dialyzed to remove sucrose with 20-kDa molecular weight cutoff (MWCO) Amicon filters and concentrated with 100-kDa MWCO centrifugal filter. GraFix cross-linked samples were also tested on a Superose 6 5/150 gel filtration column (fig. S1C) and ran on SDS-PAGE showing the fully cross-linked kinesin (fig. S1D). The cross-linked samples were analyzed for their average masses using a Refeyn Mass Photometer (Refeyn Ltd.), revealing a mass consistent with pure heterotetramers (fig. S1E), and were further used to curate sample showing oligomers (fig. S1F).

Quantifoil R1.2/1.3, 300-mesh Au grids were plasma cleaned with H_2_/O_2_, 50 W, for 30 s, and Vitrobot blotting conditions were set at 4°C and 100% humidity. KIF5B-mScarlet-KLC1 sample with Fluorinated Octyl Maltoside (FOM) detergent [0.12× to 1× critical micelle concentration (CMC)] (VitroEase, Thermo Fisher) at 3 mg/ml was pipetted to the grids, blotted 3 to 3.5 s, and plunge frozen in liquid ethane.

For cryo-EM dataset collection, G2 Krios microscope (Thermo Fisher) at 300 kV equipped with Falcon IV detector (Thermo Fisher) at ×130,000 magnification was set, and data acquisition was performed using Leginon (*60*) with beam-image shift (*61*), 7-s exposure, a total dose of ~48 e^−^/Å^2^, a pixel size of 0.926 Å, defocus ranging from −0.8 to −2.5 μm, ×130,000 magnification, and filtering through a Selectris 20-eV energy filter. A total of four datasets comprising 55,601 movies were saved as electron-event representation (EER) (*62*) files at image dimensions of 4096 pixels by 4096 pixels.

### Cryo-EM structure determination

Each EER-movie dataset was imported into RELION (*63*) separately and motion corrected with an EER fractionation of 32 using RELION’s own implementation with B-factor of 150 and patches of 5 × 5 with no binning. Contrast Transfer function (CTF) estimation was done using CTFFIND-4.1 (*64*). Initial picking was done using Laplacian of Gaussian in RELION with a 100 × 450 diameter and particle images extracted in 200-pixel box at 3.704 Å per pixel, followed by improved picking using negative-stain map and Topaz. Multiple 2D, 3D classification, and 3D refinement steps helped to remove junk, broken, or open kinesin particles leading to 36-nm comet-shaped lambda particle structures from each of the datasets in which the particle has a wide globular organization at one end and an elongated narrow region at the other end. Iterative, 2D classifications were done in cryoSPARC (*65*), particles were reextracted in RELION (*63*) and reclassified in cryoSPARC (*65*), and ~4 million particles were repicked with Topaz (*66*) in RELION (*63*). To accurately center these elongated lambda particles, we used an iterative strategy of picking particles, followed by *T* = 0 classification (*67*) (fig. S2). Aligned particles were iteratively refined, classified, and polished (fig. S3) to accumulate 664,938 particles extracted to 1.852 Å per pixel. Global consensus 3D-refined structures were determined using iterative 3D refinement and 3D classification in large box formats. Density for the subregions of the consensus cryo-EM map was further improved by recentering, reextraction, and local refinement leading to 5.6- to 8.6-Å resolution (figs. S3 and S4). Recentering and reextraction into 150-pixel box with 1.852 Å per pixel, followed by local refinement, were used for TPR-A, part of CC1^KHC^ and CC4, and KLC-CCs with 595,495 particles. 3D classification without alignment showed single class with 103,108 particles, in which both motor are in closed conformation, which were then 3DFlex (*68*) and locally refined in cryoSPARC (*65*) to resolve the motor-tail domain interaction with mScarlets-KLC TPR-B (fig. S3). These refined subregional maps were combined by overlaying onto the consensus map, leading to full kinesin lambda particle density map to 5.6- to 8.6-Å resolution throughout the structure. A model was placed into locally refined maps (CC1–CC4, motors, and KLCs) and a 36-nm flex-refined map, generated initially from 26,000 (CC2–CC3), were used to produce a 15-Å lowpass-filtered map in EMAN SBGrid software packages (*69*). This map was then used to align the CC2–CC3 “elbow” turnaround region, followed by a 60-Å lowpass filtering, leading 12-Å map. This was followed by 3D classification, refinement, and 3D variability analysis, which separated 30,746 particles that were subsequently locally refined using a 300-pixel box at 1.852 Å/pixel. Local resolutions were estimated using PHENIX (*70*) (fig. S3 and movie S1). Cryo-EM data collection, single particle data processing, and final model statistics are shown in Table 1.

**Table 1.** Cryo-EM data collection, structure determination, and model building statistics. FSC, Fourier Shell Correlation.

Parameters	Motor-B–CC0–TPR-B	Motor-A–tails	TPR-A–central section	Coiled-coil elbow region
Magnification	×130,000			
Voltage (kV)	300			
Electron exposure (e^−^/Å^2^)	48			
Defocus range (μm)	−0.8 to −2.5			
Pixel size (Å)	0.926			
Symmetry imposed	C1			
No. of final particle images	103,108	103,108	595,495	30,746
Map resolution FSC threshold (Å)	7.6	6.5	5.6	8.6
Initial model used	AlphaFold3 predictions			
Model resolution FSC threshold (Å)	0.5			
Model resolution (Å)	8			
Model composition				
Chains	4			
Nonhydrogen atoms	27,825			
Protein	3502			
Nucleic acid	0			
Ligand	0			
Root mean square deviations				
Bonds (Å)	0.002			
Angles (°)	0.539			
MolProbity score	2.20			
Clashscore	17.25			
Poor rotamers (%)	0.03			
Ramachandran plot				
Favored (%)	92.64			
Allowed (%)	7.30			
Disallowed (%)	0.06			

### XL-MS of kinesin assemblies

Kinesin KIF5B-mScarlet-KLC1 heterotetramer was cross-linked with 2 mM BS3 overnight on ice, under similar conditions to those used for cryo-EM (*39*). The cross-linked samples were then denatured (8 M urea), reduced (5 mM DTT), alkylated (15 mM indole-3-acetic acid), quenched with DTT, digested with LysC [1:100 (w/w) ratio], diluted fourfold, digested with trypsin [1:50 (w/w) ratio], desalted (Sep-Pak C18), and vacuum dried. The sample was analyzed using an UltiMate3000 UHPLC system coupled to a Q-Exactive HF-X Orbitrap. Peptides were resuspended and loaded onto PepMap 100 C18 column with solvent A (0.1% formic acid in water) and solvent B [0.1% formic acid in 98% Acetonitrile (ACN)]. Then, the peptides were separated using PepMap RSLC C18 column with a linear gradient from 5 to 25% solvent B over 100 min, then to 45% over 25 min, and to 90% over 5 min. For liquid chromatography–tandem mass spectrometry data acquisition, the Q-Exactive HF-X performed MS1 scans at 120,000 resolution [350 to 1500 mass/charge ratio (*m*/*z*)], AGC target of 3 × 10^6^, and 50-ms max injection time (IT). The top 10 precursors (*z* = 3 to 8) were isolated (1.4 *m*/*z* window) and fragmented using stepped Normalized Collision Energy (NCE) (32 ± 3). MS2 scans were at 60,000 resolution (200 to 2000 *m*/*z*), AGC target of 1 × 10^5^, and 150-ms max IT. Dynamic exclusion was set to 45 s and in-source Collision-Induced Dissociation (CID) at 10 eV.

RAW files were converted and recalibrated using the xiSEARCH preprocessing pipeline Python script. The recalibrated MGF file was searched with xiSEARCH 1.8.9 for cross-linked peptides. Search parameters were used as following: MS1 mass tolerance, 6 parts per million (ppm); MS2 mass tolerance, 10 ppm; allowed maximum number of missed cleavages, 4; minimum peptide length, 6. Cross-links were searched on the basis of the modifications at Lys and N terminus (preferred), and modifications at Ser, Thr, and Tyr were also allowed with lower priority. Carbamidomethylations (+57.021464 Da) on Cys were enforced as fixed modifications, and oxidations (+15.99491463 Da) on Met and phosphorylations on Ser/Thr/Tyr (Sp, +166.9984 Da; Tp, +181.0140 Da; Yp, +243.0269 Da) were allowed as variable modifications. FASTA file containing KIF5B, KLC1, and mScarlet was applied in the search separately. Search results were filtered in xiFDR 2.3.10 at the residue pair level to a false discovery rate of 5%. The boost function was enabled between residue pairs, and the rest of the settings was default. The mzid file generated from xiFDR was uploaded onto xiVIEW, and Protein Data Bank (PDB) file generated from cryo-EM study was imported for cross-link visualization. Figure 3 (C to I) and fig. S10 (B and D) were generated by exporting the filtered cross-links from xiVIEW and rendered with the cryo-EM ribbon model in ChimeraX (*71*) with XMAS (*72*). The connectogram in fig. S10C was generated with xiVIEW without filtration. The mass spectrometry proteomics data have been deposited to the ProteomeXchange Consortium via the PRIDE partner repository with the dataset identifier PXD073177 and 10.6019/PXD073177 and provide a table of all unique cross-links in data S1.

### Model building and XL-MS data visualization

Local refined subregional maps were aligned, and a ChimeraX (*71*) vop map was created for full-length Kinesin heterotetramer (fig. S4, A to D). Step-by-step detailed model building efforts are shown in figs. S5 and S6. AlphaFold3-predicted KIF5B-mScar-KLC1 local folded regions were used to place motor domains, coiled coils, TPRs, and mScarlet β barrel fold (figs. S4, E and F, and S9). The CC0 dimer, CC2^KHC^-CC3 elbow region, coiled coils for KHC and KLC, and C-terminal tail of KHCs and KLCs were modeled manually (figs. S5 and S6). All placed models were real-space refined in Phenix and validated using MolProbity (*70*, *73*).

### AlphaFold3 model predictions

To determine kinesin models, sequences for two copies of the KIF5B-mScarlet and KLC1 molecules were entered into a single multisubunit determination using the AlphaFold3 server (www.alphafoldserver.com) (*55*). Moderate- to high-confidence pLDDT values per residue are displayed (fig. S9A) and their corresponding Predicted Aligned Error (PAE) matrix with accuracy of residue position error. Five AlphaFold3 models (fig. S9B) reveal highly variable positions in KLC-TPR domains and KHC tail domains and their associated mScarlets. To determine KIF5B-MAP7D3^CT^ AlphaFold3 models, sequences for two copies of KIF5B (residues 1 to 587) MAP7D3^CT^ (residues 476 to 876) were entered into a single multisubunit determination using the AlphaFold3 server. A single representative model is presented in fig. S14A, with the moderate- to high-confidence pLDDT values per residue displayed.

### TIRF assays

TIRF flow chambers were assembled from acid-washed glass coverslips, precleaned slides (Thermo Fisher), and double-sided tape. Chambers were functionalized by sequential incubation with poly-l-lysine (PLL)–polyethylene glycol–biotin (0.5 mg/ml; Surface Solutions) for 5 to 10 min and streptavidin (0.5 mg/ml; Thermo Fisher) for 5 min.

Taxol-stabilized MTs (10 μM) in BRB80 buffer [80 mM Pipes (pH 6.8), 1 mM MgCl_2_, and 1 mM EGTA] were flowed into the chamber and incubated for 2 to 5 min to allow immobilization. Unbound MTs were removed by washing with SRP90 assay buffer [90 mM Hepes, 50 mM KCH_3_COO, 2 mM Mg(CH_3_COO)_2_, 1 mM EGTA, and 10% glycerol (pH 7.6)] supplemented with bovine serum albumin (BSA; 1 mg/ml), biotin-BSA (0.05 mg/ml), K-casein (0.2 mg/ml), 0.5% Pluronic F-127, 10 μM taxol, and 1 μM phalloidin. Last, kinesin-1 (KIF5B-KLC1) at the indicated concentrations, with or without MAP7D3^CT^, was introduced in SRP90 assay buffer containing 2 mM Mg-ATP (Sigma-Aldrich).

All imaging was performed on a Nikon TE microscope equipped with a PlanApo 100×/1.49 numerical aperture objective, a TIRF illuminator (LU-N4), and an Andor iXon EMCCD camera, controlled with MicroManager 1.4 software (*74*). Data were analyzed manually using kymographs generated in ImageJ (Fiji).
